# TUXEDO-3: A phase 2 trial of patritumab deruxtecan for patients with leptomeningeal metastasis

**DOI:** 10.1093/neuonc/noaf122

**Published:** 2025-06-04

**Authors:** Emilie Le Rhun, Enrico Franceschi, Michael Weller

**Affiliations:** Department of Medical Oncology and Hematology, Brain Tumor Center, University Hospital and University of Zurich, Zurich, Switzerland; Department of Nervous System Medical Oncology, IRCCS Istituto delle Scienze Neurologiche di Bologna, Bologna, Italy; Department of Neurology, Brain Tumor Center, University Hospital and University of Zurich, Zurich, Switzerland

The international single arm phase II TUXEDO-3 trial (NCT05865990) investigated the central nervous system (CNS) activity of the antibody drug conjugate, patritumab deruxtecan (HER3-DXd), a fully human anti-HER3 IgG1 antibody attached to topoisomerase I inhibitor payload *via* a tetrapeptide-based cleavable linked with a drug-to-antibody ratio of 8:1. Cohort 3, recently published in *Nature Medicine*^1^ explored the efficacy and the safety of HER3-DXd in patients with leptomeningeal metastasis from any tumor type. Human epidermal growth factor receptor 3 (HER3/ErbB3) is a tyrosine kinase receptor with oncogenic properties, including promotion of tumor growth, invasion, and resistance to therapy. HER3 has been shown to be overexpressed in brain metastases from breast and non-small lung cancer (NSCLC).^2^ In a phase I/II trial in patients with metastatic breast cancer, 2 recommended expansion doses (4.8 mg/kg and 6.4 mg/kg) of HER3-DXd were proposed.^3^ In a phase II study in patients with Epidermal Growth Factor Receptor (EGFR)-mutant NSCLC, other doses were explored. The trial had a dose titration arm from 3.3 to 6.4 mg/kg closed at 51 patients, and a fixed dose arm at 5.6 mg/kg which enrolled 226 patients.^4^ In the breast cancer study, grade 3 or more treatment-emergent adverse events (TEAE) were noted in 130 patients (71.4%); 18 patients (9.9%) discontinued treatment because of TEAE. In the NSCLC study, TEAEs were noted in 31 patients (62%) in the titration arm and in 146 patients (64.9%) in the fixed dose arm; 5 patients (10%) in the titration arm and 16 patients (7.1%) interrupted treatment because of TEAE. Toxicity was mainly hematologic and gastrointestinal. In both studies, responses were reported, and several phase II and III trials are ongoing.^3,4^

The TUXEDO-3 trial had a stage 2 design. In stage I, 10 evaluable patients had to be enrolled, with the objective to continue the study if more than 1 patient was still alive at 3 months. In stage II, the objective was to observe 3 or more of 10 patients alive at 3 months. Secondary endpoints included local assessment of the overall response rate per *Response Assessment in Neuro-Oncology* (RANO) or RECIST criteria, safety and toxicity per CTCAE 5.0, quality of life (EORTC QLQ C30 and BN20), and neurological function (NANO). Adult patients with an Eastern Cooperative Oncology Group performance status between 0 and 2, a diagnosis of leptomeningeal metastasis from any type of solid tumor, newly diagnosed, or progressing after radiotherapy, could be included. Patients with previous anti-HER3 therapy were excluded, but, importantly, HER3 expression was not required for inclusion.

Twenty evaluable patients with newly diagnosed leptomeningeal metastasis (18 female patients, median age 51.5), including 9 patients with tumor cells present in the cerebrospinal fluid, were enrolled in 7 months across 8 centers. Cerebrospinal fluid (CSF) data for the other 11 patients were not reported. Primary tumors were breast cancer (*N* = 12, 60%), lung cancer (*N* = 6, 30%), melanoma (*N* = 1, 5%), and ovarian cancer (*N* = 1, 5%). Patients had a median of 2 previous lines of anti-cancer treatment (range 0–6). Patients received HER3-Dxd 5.6 mg/kg intravenously every 3 weeks. The median follow-up was 5.4 months (range 0.8–12.0), and 4 patients were still on treatment at the time of the analysis. The median treatment duration was 3.4 months (range 0.0–11.5).

Median survival was 10.5 months (95% confidence interval [CI] 2.1–not achieved [NA]), and the 3-month (primary endpoint) and 6-month overall survival rates were 69.6% (95% CI 44.5–85.1) and 58.9% (95% CI 34.3–77.0) months. Twelve patients (60%) showed neurologic symptoms or signs at baseline. At the time of the analysis, 2 patients (10%) had a clinical response per NANO scale, 11 patients (55%) had stable disease, and 7 patients had no follow-up assessment. The overall response rate was 11.1% (2/18 patients) for brain metastases per RANO-brain metastases local assessment, and 30.8% (4/13 patients) for extra-CNS response per RECIST 1.1. Median time to intracranial response was 1.8 months (95% CI: 1.6–1.9) months, and to extracranial response 2.0 months (95% CI 1.9–2.1). Median progression-free survival for intracranial disease was 9.9 months (95% CI 1.6–NA, 9/20), and for extracranial disease, 6.8 months (95% CI 2.1–NA, 10/20). No correlation was observed between HER3 expression on 15 samples (including 2 brain metastases and 13 extra-CNS sites) and outcome.

Grade 3 or more TEAEs were noted in 15 patients (68.2%), and 1 patient discontinued treatment because of TEAE. A dose reduction of HER3-DXd due to TEAE was instituted in 2 patients, and a permanent discontinuation occurred in 1 patient. Most common TEAE included anemia (9 patients, 40.1%), nausea (7 patients, 31.8%), neutropenia (6 patients, 27.3%), diarrhea (6 patients, 27.3%), and fatigue (6 patients, 27.3%), many of which are not infrequent in this patient population.

This study reports a 3-month overall survival of 69.6% and a median overall survival of 10.5 months. The toxicity profile was consistent with previous studies.^3,4^ The median overall survival, the preferred endpoint for systemic agents in patients with leptomeningeal metastasis, is promising. Further studies are now required to confirm the results, considering potential limitations of the TUXEDO-3 trial, with a larger sample size per tumor entity, ideally molecularly defined, as the prognosis and responsiveness may vary. Standardized diagnostic work-up, including clinical assessment, neuroimaging, and CSF studies, should be included.^5^ Other uncontrolled studies with systemic agents have reported survival between 8 and 12 months with ANG1005 (no CSF cytology available), abemaciclib (2/10—20% patients with tumor cells in the CSF), and tucatinib (5/17—39% patients with tumor cells in the CSF).^6^ Of note, studies enrolling only patients with tumor cells in the CSF have reported poorer survival rates in general (intravenous pembrolizumab regimen, median overall survival [OS]: 3.6 months; bevacizumab, cisplatin, and etoposide regimen, median OS: 4.7 months).^6^ In contrast, a median survival of 18.8 months was reported in patients with leptomeningeal metastasis (no CSF cytology available, brain imaging could be computed tomography or magnetic resonance imaging) from EGFR-positive NSCLC treated with osimertinib at 160 mg once daily.^7^ Accordingly, patient selection will heavily impact on outcomes in uncontrolled trials for patients with leptomeningeal metastasis, notably if an endpoint of overall survival at 3 months is selected.

The TUXEDO-3 study provides proof of concept that responses in the brain can be achieved with HER3-DXd. However, no formal response assessment of leptomeningeal metastasis was foreseen, and the timing of examination during the follow-up was left at the discretion of the investigator during the follow-up and had to be performed if clinically indicated or in case of suspicion of progression, or start of a new anti-cancer treatment. The authors justified the absence of leptomeningeal metastasis response assessment with its lack of reliability, but several efforts are being made to introduce response assessment of leptomeningeal metastasis for the improvement of clinical trials.^8–10^

The safety of HER3-DXd was similar in the TUXEDO-3 study as in previous studies. Alternative dosing schedules to achieve better coverage of the CNS space could be explored. These preliminary promising results call for follow-up, ideally in a randomized phase II setting, for example, versus best physician’s choice, and for improved biomarker studies, for example, quantitative assessment of HER3 expression in larger cohorts, expression of other ErbB family dimerization partners or ligands. Future studies might also focus on the best opportunities to combine HER3-DXd with other pillars of treatment of leptomeningeal metastasis, such as intrathecal pharmacotherapy and radiotherapy.
